# Hydrogen-rich water combined with traditional Chinese medicine compound in the treatment of kidney stones: a randomized controlled prospective clinical trial

**DOI:** 10.4103/mgr.MEDGASRES-D-26-00067

**Published:** 2026-09-12

**Authors:** Yu Yin, Jing Wang, Feng Lian, Yunqing Tang, Yidi Shen, Yue Guo, Qiming Xu, Jing Hu, Guobin Yang, Hongtao Lu, Wenrui Liu

**Affiliations:** 1 Department of Nephrology, Seventh People’s Hospital Affiliated to Shanghai University of Traditional Chinese Medicine, Shanghai, China; 2 Department of Naval Medicine, Naval Medical University, Shanghai, China; 3 Department of Traditional Chinese Medicine (TCM) Geriatrics, Huadong Hospital Affiliated to Fudan University, Shanghai, China; 4 Department of Ultrasound Medicine, Seventh People’s Hospital Affiliated to Shanghai University of Traditional Chinese Medicine, Shanghai, China

**Keywords:** blood uric acid, glutathione reductase, hydrogen-rich water, inflammation, kidney stone, oxidative stress, renal tubules, traditional Chinese medicine, uric metabolism, *Ye-Shi-Shi-Lin*-Formula

## Abstract

The formation and development of kidney stones are related to abnormal urine metabolism, oxidative stress and inflammation. Hydrogen-rich water has clear efficacy in antioxidant and improving inflammatory status, while *Ye-Shi-Shi-Lin*-Formula is a clinically effective traditional Chinese medicine compound preparation for treating kidney stones. This randomized controlled prospective clinical trial from July 2025 to March 2026 at the Seventh People’s Hospital Affiliated to Shanghai University of Traditional Chinese Medicine examined the effect of hydrogen-rich water and *Ye-Shi-Shi-Lin*-Formula on kidney stones. The included 100 patients with kidney stones were randomly divided into blank, hydrogen-rich water, hydrogen-rich water and Chinese medicine, and Chinese medicines groups. The patients received drinking hydrogen-rich water and/or *Ye-Shi-Shi-Lin*-Formula daily for 12 weeks. All subjects received basic treatment following the guidelines. Imaging examination, urine metabolism testing, renal function, inflammation and oxidative stress index, blood routine and liver function are used to detect the efficacy and safety of hydrogen-rich water and *Ye-Shi-Shi-Lin*-Formula. Results showed that the total effective rate of kidney stone treatment in blank group, Chinese medicines group, hydrogen-rich water and Chinese medicine group and hydrogen-rich water group was 28%, 76%, 80% and 32%. The *Ye-Shi-Shi-Lin*-Formula can partially alleviate the oxidative stress, uric acid metabolism and urinary magnesium levels of patients with kidney stone and improve the function of renal tubules. The hydrogen-rich water therapy showed only efficacy in improving glutathione reductase but the combination of hydrogen-rich water and *Ye-Shi-Shi-Lin*-Formula has shown superior efficacy in improving oxidative stress and related metabolic factors of blood uric acid and urine stones, as well as in renal tubular function. The results indicate that the addition of hydrogen-rich water can improve urinary metabolism and oxidative stress status in the treatment of kidney stones with *Ye-Shi-Shi-Lin*-Formula. The study was registered at the International Traditional Medicine Clinical Trial Registry (Registration No. ITMCTR2025001446).

## Graphical abstract

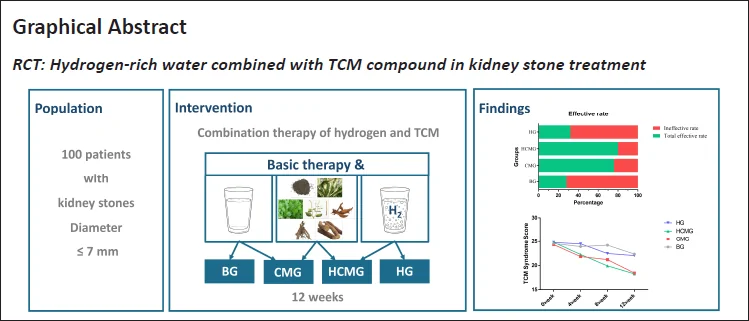

## Introduction

Kidney stones are the most common health condition affecting the kidney and urinary tract, and is a systemic disease associated with metabolic syndrome,^1^ increasing the risk of chronic kidney disease.^2^ Regardless of obstruction, renal interstitial fibrosis and renal dysfunction caused by kidney stones continue to progress,^3^ leading to end-stage renal disease. The solute supersaturation of urine causes crystal formation,^4^ while both supersaturated solutes and formed crystals can act on the epithelial cells of the renal tubules, stimulating reactive oxygen species (ROS) and inflammation, leading to tubulointerstitial inflammation and interstitial fibrosis, triggering a cascade reaction and ultimately forming kidney stones. The dissolution and prevention of the stone relapse are the main focuses of urolithiasis treatment.^5^ At present, treatments for kidney stones mainly include drinking plenty of water, taking drugs to treat small stones, improving recovery after surgery and helping prevent stone recurrence.^6^ However, water and current drugs typically cannot prevent stone formation and recurrence, and most patients ultimately need surgery. After the stones are removed, the existing kidney damage cannot be reversed.

Herbal medicines have a rich global history in the treatment of nephrolithiasis,^7^ and offer a safer alternative treatment strategy. In contrast to current surgical and pharmaceutical treatments, herbal therapy is regarded as an option that can improve patient prognosis and shows potential for preventing stone recurrence, presenting fewer side effects.^8^ Traditional Chinese medicinal (TCM) plants provide a rich resource pool for global drug research and development.^9^,^10^ *Ye-Shi-Shi-Lin*-Formula (YSSLF) is a traditional Chinese medicinal prescription based on the clinical experience of Professor Jinghua Ye in the treatment of kidney stones. In previous clinical practices, it has been proven to have a favorable effect on the treatment of small kidney stones and the prevention of recurrence. YSSLF is composed of Glechoma longituba, Plantain Seed, Pyrrosia, Achyranthes bidentata Blume, White Peony, Radix Pueraria and Prepared Glycyrrhiza. A previous study has confirmed the antioxidant and anti-inflammatory effects of Glechoma longituba in kidney stone treatment.^11^

Hydrogen (H_2_) is the simplest and most abundant element in nature, and it was once believed to have no biological effects on higher organisms. H_2_ was first reported to be used in diving medicine,^12^ and it has been found to exhibit preventive and therapeutic effects in various diseases now. Due to the safety of H_2_ gas to the human body, its potential clinical application prospects have been widely valued. In recent years, H_2_ has been suggested as a potential therapeutic and preventive treatment for various kidney diseases.^13^ ROS play an important role in the formation of calcium oxalate stones.^14^ While molecular H_2_ also exerts multiple biological effects through anti-inflammatory and antioxidant.^15^ Therefore, the efficacy of the combination of YSSLF and H_2_ in the treatment of kidney stones should be explored.

This study aims to use a randomized controlled prospective research scheme through the comparison of four groups to observe the changes of clinical symptoms and laboratory test indicators of patients with kidney stones treated by YSSLF combined with H_2_-rich water (HRW) before and after treatment to summarize the clinical efficacy and safety of YSSLF combined with HRW to identify abnormal urinary metabolism during the course of kidney stone disease and to explore the metabolic mechanism of YSSLF combined with HRW in treating kidney stones.

## Methods

This randomized controlled trial was registered at the International Traditional Medicine Clinical Trial Registry (https://itmctr.ccebtcm.org.cn/; Registration No. ITMCTR2025001446) on July 20, 2025. The present study was conducted in accordance with the principles outlined in the *Declaration of Helsinki* and was approved by the Ethics Committee of Shanghai Seventh People’s Hospital (ethics approval No. 2025-7th-HIRB-035) on February 28, 2025. Written informed consent was obtained from the patients and the guardians of the patients who participated in the study. The study was performed in accordance with the CONsolidated Standards Of Reporting Trials (CONSORT) Guideline^16^ (**Additional file 1**).

**Table:** CONSORT 2025 checklist of information to include when reporting a randomised trial*

Section / Topic	No	CONSORT 2025 checklist item description	Reported on page no.
**Title and abstract**			
Title and structured abstract	1a	Identification as a randomised trial	Page 1
	1b	Structured summary of the trial design, methods, results, and conclusions	Page 1
**Open science**			
Trial registration	2	Name of trial registry, identifying number (with URL) and date of registration	Page 2
Protocol and statistical analysis plan	3	Where the trial protocol and statistical analysis plan can be accessed	Page 3
Data sharing	4	Where and how the individual de-identified participant data (including data dictionary), statistical code and any other materials can be accessed	inapplicable
Funding and conflicts of interest	5a	Sources of funding and other support (e.g., supply of drugs), and role of funders in the design, conduct, analysis and reporting of the trial	Page 1
	5b	Financial and other conflicts of interest of the manuscript authors	Page 1
**Introduction**			
Background and rationale	6	Scientific background and rationale	Page 1
Objectives	7	Specific objectives related to benefits and harms	Page 1
**Methods**			
Patient and public involvement	8	Details of patient or public involvement in the design, conduct and reporting of the trial	Page 2
Trial design	9	Description of trial design including type of trial (e.g., parallel group, crossover), allocation ratio, and framework (e.g., superiority, equivalence, non-inferiority, exploratory)	Page 2-3
Changes to trial protocol	10	Important changes to the trial after it commenced including any outcomes or analyses that were not prespecified, with reason	inapplicable
Trial setting	11	Settings (e.g., community, hospital) and locations (e.g., countries, sites) where the trial was conducted	Page 2
Eligibility criteria	12a	Eligibility criteria for participants	Page 2
	12b	If applicable, eligibility criteria for sites and for individuals delivering the interventions (e.g., surgeons, physiotherapists)	Page 2
Intervention and comparator	13	Intervention and comparator with sufficient details to allow replication. If relevant, where additional materials describing the intervention and comparator (e.g., intervention manual) can be accessed	Page 2
Outcomes	14	Pre-specified primary and secondary outcomes, including the specific measurement variable (e.g., systolic blood pressure), analysis metric (e.g., change from baseline, final value, time to event), method of aggregation (e.g., median, proportion), and time point for each outcome	Page 2-3
Harms	15	How harms were defined and assessed (e.g., systematically, non-systematically)	Page 3
Sample size	16a	How sample size was determined, including all assumptions supporting the sample size calculation	Page 3
	16b	Explanation of any interim analyses and stopping guidelines	inapplicable
Randomisation:			
Sequence generation	17a	Who generated the random allocation sequence and the method used	Page 2
	17b	Type of randomisation and details of any restriction (e.g., stratification, blocking and block size)	inapplicable
Allocation concealment mechanism	18	Mechanism used to implement the random allocation sequence (e.g., central computer/telephone; sequentially numbered, opaque, sealed containers), describing any steps to conceal the sequence until interventions were assigned	Page 2
Implementation	19	Whether the personnel who enrolled and those who assigned participants to the interventions had access to the random allocation sequence	Page 2
Blinding	20a	Who was blinded after assignment to interventions (e.g., participants, care providers, outcome assessors, data analysts)	Page 2
	20b	If blinded, how blinding was achieved and description of the similarity of interventions	Page 2
Statistical methods	21a	Statistical methods used to compare groups for primary and secondary outcomes, including harms	Page 3
	21b	Definition of who is included in each analysis (e.g., all randomised participants), and in which group	Page 3
	21c	How missing data were handled in the analysis	inapplicable
	21d	Methods for any additional analyses (e.g., subgroup and sensitivity analyses), distinguishing prespecified from post-hoc	inapplicable
**Results**			
Participant flow, including flow diagram	22a	For each group, the numbers of participants who were randomly assigned, received intended intervention, and were analysed for the primary outcome	Page 3-4
	22b	For each group, losses and exclusions after randomisation, together with reasons	Page 4
Recruitment	23a	Dates defining the periods of recruitment and follow-up for outcomes of benefits and harms	Page 2
	23b	If relevant, why the trial ended or was stopped	inapplicable
Intervention and comparator delivery	24a	Intervention and comparator as they were actually administered (e.g., where appropriate, who delivered the intervention/comparator, how participants adhered, whether they were delivered as intended [fidelity])	Page 2-4
	24b	Concomitant care received during the trial for each group	Page 2
Baseline data	25	A table showing baseline demographic and clinical characteristics for each group	Table 2
Numbers analysed, outcomes and estimation	26	For each primary and secondary outcome, by group: • the number of participants included in the analysis • the number of participants with available data at the outcome time point • result for each group, and the estimated effect size and its precision (such as 95% confidence interval) • for binary outcomes, presentation of both absolute and relative effect size	Pag4-6. Table 2-6 Additional Table 1-
Harms	27	All harms or unintended events in each group	Page 2
Ancillary analyses	28	Any other analyses performed, including subgroup and sensitivity analyses, distinguishing pre-specified from post-hoc	inapplicable
**Discussion**			
Interpretation	29	Interpretation consistent with results, balancing benefits and harms, and considering other relevant evidence	Page 6
Limitations	30	Trial limitations, addressing sources of potential bias, imprecision, generalisability, and, if relevant, multiplicity of analyses	Page 6

### Subjects

This study enrolled a total of 100 patients with kidney stones at the Shanghai Seventh People’s Hospital between July 2025 and March 2026.

The inclusion criteria for participation in the study were as follows: (1) Patients who met the diagnostic criteria of Western medicine for kidney stones, (2) patients who met the criteria of traditional chinese medicinal syndrome differentiation, (3) the age ranged from 18 to 65 years old, (4) renal stone diameter ≤ 7 mm, (5) the renal function of the affected side is good, (6) if there is hydronephrosis on the affected side, the degree of hydronephrosis should be moderate or below, and (7) patients willing to receive traditional Chinese medicine treatment and had normal activity ability and good compliance.

Patients who experienced any of the following events were also excluded: (1) Pregnant and lactating women female individuals with positive pregnancy test, (2) those with malformations stenosis obstruction surgical scar adhesions etc. in the kidneys and ureters, (3) those with severe primary diseases such as coronary heart disease uremia leukemia etc. or known serious diseases that affect their survival (such as tumors or AIDS) or patients with mental or legal disabilities, (4) those who have used medication to treat urinary tract stones within the past week, (5) individuals with various infections within a month or those with persistent high blood pressure and blood sugar that are difficult to control with medication, (6) those with severe anemia acid-base balance disorders and electrolyte imbalances and other complications, (7) other lesions that according to the researcher’s judgment reduce the possibility of inclusion or make the inclusion process complicated such as situations that may cause loss to follow-up due to frequent changes in working environment, (8) those suspected or confirmed to have a history of alcohol or drug abuse, (9) allergic constitution such as those with a history of allergies to two or more drugs or foods or those who are known to be allergic to the intervention drugs in this study, and (10) patients who are currently participating in clinical trials of other drugs.

### Randomized grouping and blinding

Randomization was performed by an independent statistician using a computer-generated random number sequence. The research project leader employed a computerized randomization procedure to assign 100 numbers (1–100) into four groups of 25, which were documented on a grouping form. The researchers from the outpatient clinic and inpatient ward of the nephrology department handled participant recruitment. The project leader assigned each recruited participant to a group based on the corresponding random number recorded on the grouping form and communicated this grouping to the researcher prior to the commencement of treatment.

The enrolled patients were assigned to four groups: blank group, which received basic treatment combined with drinking regular water; HRW group, which received basic treatment combined with drinking HRW; HRW + Chinese medicine group, which received basic treatment, drinking HRW combined with drinking YSSLF; and Chinese medicine group, which received basic treatment, drinking regular water combined with drinking YSSLF. The nephrologist then administered different therapeutic measures according to the intervention protocol for the assigned subgroup. The researchers who conducted the follow-up observations and blood sample testing were blinded to the groups.

### Preparation and administration of the *Ye-Shi-Shi-Lin*-Formula

YSSLF is composed of seven herbs (**Table 1**). For the above medications, the first decoction is prepared by adding 250 mL of water, boiling over high heat until it boils, and then simmering over low heat for 30 minutes. The resulting liquid is 100 mL. The second decoction is made by adding 200 mL of water, boiling for 15 minutes, and then taking 100 mL of the liquid. The 100 mL of each decoction is mixed, and the mixture is taken orally twice in the morning and evening.

**Table 1:** Composion of *Ye-Shi-Shi-Lin*-Formula

Chinese name	Botanical name	Latin name	English name	Medical part	Weight (g)	Category
*Lianqiancao*	Glechoma longituba	*Glechoma longituba (Nakai)* Kupr.	Herba Glechomae	Whole herbs	30	Chief
*Cheqianzi*	Plantain seed	*Plantago asiatica* L. *or Plantagodepressa Willd.*	Semen Plantaginis	Seed	30	Chief
*Shiwei*	Pyrrosia	*Pyrrosia lingua (Thunb.)* Farw.	Pyrrosia lingua	Whole herbs	30	Chief
*Niuxi*	Achyranthes bidentata Blume	*Achyranthis Bidentatae Radix*	Twotooth Achyranthes Root	Root	10	Deputy
*Baishao*	White Peony	*Paeonia lactiflora* Pall.	Radix Paeoniae Alba	Root	15	Assistant
*Gegen*	Lobed Kudzuvine root	*Pueraria montana (Lour.)* Merr.	Radix Pueraria	Root	30	Assistant
*Zhigancao*	Prepared Glycyrrhiza	*Glycyrrhiza uralensis* Fisch*., Glycyrrhiza glabra* L. *or Glycyrrhiza inflata* Bat.	Radix Glycyrrhizae Preparata	Root and stem	6	Assistant

### Consumption of hydrogen-rich water

HRW was produced by electrolyzing water using the NB-B81 multifunctional H_2_ molecule generator from Shanghai Nanobubble Nanotechnology Co., Ltd. (Shanghai, China). The equipment has a capacity of 280 mL and the hydrogen concentration in hydrogen water is 1–1.5 ppm. Before the trial, the H_2_ water cup was provided to each participant to ensure they drank 2 L of HRW daily (if the participants sent four cups of drinking water videos per day, it was considered as meeting the standard; if not, it was considered as not meeting the standard, then a phone reminder was given immediately) and the process lasted for 12 weeks.

### Basic therapy

The basic treatment primarily involves dietary nutrition therapy, which focuses on avoiding diets high in calcium, oxalic acid, and uric acid. Treatment with drinking regular water: before the start of the trial, a 500-mL capacity water cup was provided to ensure that the participants drank 2 L of pure water daily (The mode of reminder and supervision is the same as HRW) and the process lasted for 12 weeks.

### Data collection and processing

The basic information and diagnostic data were collected at week 0. Urological ultrasound and blood and urine collection were conducted at week 0 and 12. The general characteristics, treatment plan, concurrent medications, traditional Chinese medicinal syndromes and general conditions were collected at week 0, 4, 8 and 12.

The general characteristics, such as sex, age, body temperature, body mass index, blood pressure, heart rate, respiratory rate and waist-hip rate for each patient were recorded.

Alanine aminotransferase, uric acid and serum creatinine were detected by automatic biochemical analyzer (TBA-FX8, Canon Medical Systems (China) CO., LTD, Beijing, China) using alanine substrate and creatine oxidase method. Aspartate aminotransferase, urinary N-acetyl-β-D-glucosaminidase (NAG) and urinary β2-microglobulin (β2-MG) were detected by Clinical Analyzer (SHHT008AS, Hitachi, Tokyo, Japan) using International Federation of Clinical Chemistry and Laboratory Medicine, 6-methyl-2-pyridyl-1-thio-n-acetyl-β-D-glucosamine substrate method and immunoturbidimetry. Interleukin-6 was detected by dry fluorescence analyzer (TZ-3000, Jiangsu Realai Biotechnology Co., Ltd, Taizhou, Jiangsu, China) using dry immunofluorescence method. Glutathione reductase (GSH) and superoxide dismutase were detected by the fully automatic biochemical analyzer (HITACHI008, Hitachi) using ultraviolet enzymatic and resorcinol substrate method. Red blood cells, hemoglobin, white blood cells and platelets were measured via Sysmex XN100 Hematology Analyzer (Sysmex, Chūō-ku, Kobe, Japan) using flow cytometry + fluorescence staining, sheath impedance and sodium lauryl sulfate hemoglobin detection. Urinary H_2_ ion concentration (PH) was detected by AX4280 automatic urine analyzer (Arkray, Inc., Tokyo, Japan) using dry chemical method. Urinary calcium/creatinine, urinary oxalate/creatinine, urinary phosphate/creatinine, urinary uric acid, urinary citrate/creatinine, and urinary magnesium/creatinine were detected by CIC-D100 ion chromatograph (Chongqing Drawell Instrument Co., Ltd., Chongqing, China) and SH-CC-1 4.6 × 150 mm chromatographic column (Qingdao Shenghan Chromatograph Technology Co., Ltd, Qingdao, Shandong, China) using ion chromatography.

The primary indicator is the overall effective rate for evaluating the comprehensive therapeutic effect of kidney stone treatment, including recovery and effective. The criteria for comprehensive efficacy evaluation are as follows: Recovery: clinical symptoms and signs disappear, and discharged stones are collected; or even though discharged stones are not collected, there is a clear feeling of stone discharge during a certain urination process, and then clinical symptoms and signs disappear, ultrasound examination shows the stone and hydronephrosis disappears. Effective: clinical symptoms and signs disappear; and ultrasound examination shows that the stone passes through the first stenosis and enters the upper segment of the ureter, or the stone shrinks by ≥ 30%, or moves down by more than 3 cm, or the hydronephrosis disappear. Ineffective: clinical symptoms and signs have not disappeared, ultrasound examination shows that the stone changes < 30% of the original, or there is no improvement in renal hydronephrosis.

The secondary indicators were risk factors indicators for kidney stones in urine (urinary calcium/creatinine, oxalate/creatinine, phosphate/creatinine, uric acid, citrate/creatinine, magnesium/creatinine, urinary PH), renal injury markers in urine (urine NAG and β2-MG), clinical sign, syndrome score of traditional Chinese medicine, renal function indicator (serum creatinine), inflammatory factor (interleukin-6) and oxidative stress indicator (GSH and superoxide dismutase) in serum. Adverse events include liver function indicators (alanine aminotransferase and aspartate aminotransferase) and blood cell analysis (red blood cells, hemoglobin, white blood cells and platelets).

### Traditional Chinese medicinal syndrome score

Clinical symptoms were assessed by clinical observation tables to assess syndrome scores. The syndrome differentiation criteria were formulated as follows: Main symptoms: unilateral or bilateral lower back and abdominal pain, hematuria, frequent urination, urgency to urinate, and painful urination. Secondary symptoms: languid, pallid complexion, chest tightness or restlessness, lumbar debility, dry stool. Tongue inspection and pulse manifestation: yellow and greasy tongue coating, and there may be lingual hemorrhoid, with wiry pulse or rapid pulse. The scoring grades were generally 0, 1, 2, and 3 points for normal, mild, moderate, and severe, respectively. For symptoms without severity classification, 0 point will be given for no symptoms, and 2 points will be given for symptoms.

### Sample size calculation

We determined the sample size based on preliminary experimental results on overall effective rate in adults is 80%, 80%, 30% and 30%. The required sample size for this study was calculated by the following formula:


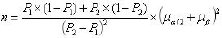


Assuming a two-sided inspection level of α = 0.05, as it is a four-group design and subsequent pairwise comparisons are involved, α is adjusted to 0.05/4 = 0.0125, and the inspection efficiency (1 – β) = 0.9. According to the table, μ_α/2_ = 2.5; μ_β_ = 1.28.

By substituting the formula, the sample size of the treatment group was calculated to be *n* = 21. To account for a potential loss to follow-up rate of 15%, we proposed including 25 patients in each group, for a total of 100 patients.

### Statistical analysis

Continuous variables were first tested for normality. The data in line with normal distribution are reported as the mean ± standard deviation (SD), the comparison between multiple groups is performed by one-way analysis of variance, the least significant difference method is used for multiple comparisons, and the intra-group comparison is performed by paired *t*-test. The measurement data or grade data that did not conform to the normal distribution are expressed as median (interquartile range), the comparison between multiple groups is performed by the Kruskal-Wallis rank sum test, and the intra-group comparison is performed by Friedman test. The counting data were expressed as the number (percentage), and the chi-square test was used for comparison between groups, and Bonferroni method was used for multiple comparisons on this basis. For traditional Chinese medicinal syndrome scores detected at multiple time points, first define their normal distribution and homogeneity of variance, then use Mauchly’s sphericity test to clarify the underlying assumptions of the data are valid, finally analyze the data by repeated measures analysis of variance to simultaneously test intra- and inter-group differences and detect interactions. The significance threshold for all comparisons was set at *P* < 0.05. Statistical analysis was performed via SPSS Statistics for Windows (Version 27.0, IBM Corp., Armonk, NY, USA) and GraphPad Prism 9 (GraphPad Software, Boston, MA, USA, www.graphpad.com).

## Results

### Baseline characteristics of enrolled participants

After screening 128 patients, 9 did not meet the inclusion criteria, 4 met the exclusion criteria, 7 declined to participate, and 2 were excluded for other reasons. Finally, 106 were enrolled (**Figure 1**), with 26 in the blank group, 27 in the Chinese medicine group, 27 in the HRW + Chinese medicine group, and 26 in the HRW group. Six patients withdraw the study: one patient in the blank group due to backache; two patients in the Chinese medicine group due to unwilling to continue and the backache; two patients in the HRW + Chinese medicine group due to diarrhea and conversion to surgery and one patient in the HRW group due to conversion to surgery. As shown in **Table 2** and **Additional Table 1**, there were no markedly differences in sex, age, and stone diameter among the patients in the four groups.

**Figure 1:**
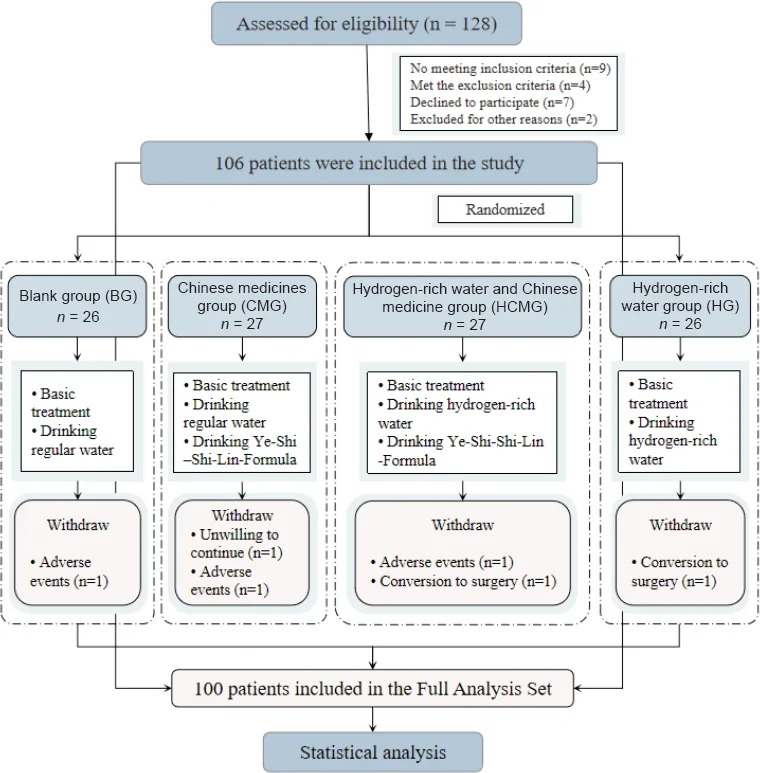
The flowchart of this study. A total of 128 patients were screened. Of these, 9 did not meet the inclusion criteria, 4 met the exclusion criteria, 7 declined to participate, and 2 were excluded for other reasons. Consequently, 106 patients were enrolled: 26 in the BG, 27 in the CMG, 27 in the HCMG, and 26 in the HG. Six patients withdrew from the study. One patient in the BG withdrew due to backache. Two patients in the CMG withdrew because they were unwilling to continue or experienced backache. Two patients in the HCMG withdrew due to diarrhea or conversion to surgery. One patient in the HG withdrew due to conversion to surgery. BG: Blank group; CMG: Chinese medicines group; HCMG: hydrogen-rich water and Chinese medicine group; HG: hydrogen-rich water group.

**Table 2:** Baseline data of patients in the four groups

Variable	BG (*n* = 25)	CMG (*n* = 25)	HCMG (*n* = 25)	HG (*n* = 25)	*χ*^2^/*F*/*H*	*P*
Male^a^	17(68%)	15(60%)	12(48%)	14(56%)	2.135	0.545
Age (yr)^b^	43.28±10.91	47.56±11.99	43.68±9.32	43.92±12.27	0.787	0.504
Stone diameter^c^	6 (4, 6)	5 (4, 6)	5 (5, 6)	5 (4, 6)	3.576	0.311

^a^ Data are expressed as number (percentage) and were analyzed by the chi-square test. ^b^ Data are expressed as the mean ± SD, and were analyzed by one-way analysis of variance followed by the least significant difference. ^c^ Data are expressed as median (interquartile range), and were analyzed by the Kruskal-Wallis rank sum test. BG: Blank group; CMG: Chinese medicines group; HCMG: hydrogen-rich water and Chinese medicine group; HG: hydrogen-rich water group.

**Additional Table 1:** General characteristics in the four groups

General characteristics	Group	BG (*n* = 25)	CMG (*n* = 25)	HCMG (*n* = 25)	HG (*n* = 25)	Intergroup comparison		*P*					
	
						F/H	*P*	BG *vs.* CMG	BG *vs.* HCMG	BG *vs.* HG	CMG *vs.* HCMG	CMG *vs.* HG	HCMG *vs.* HG
HR^a^	Before treatment	75±12	75±11	75±13	77±12	0.164	0.92	0.952	0.99	0.581	0.943	0.541	0.59
	After treatment	77±13	72±11	74±11	74±11	0.81	0.492	0.144	0.262	0.295	0.729	0.675	0.941
Intra-group comparison^c^	*T*	-0.673	0.59	0.489	0.884								
	*P*	0.507	0.561	0.629	0.385								
SBP^a^	Before treatment	114.2±9.7	116.8±9.5	118.5±9.2	117.6±11.0	0.787	0.504	0.133	0.346	0.477	0.571	0.425	0.816
	After treatment	118.4±9.4	116.2±10.2	116.9±9.8	114.4±10.1	0.77	0.513	0.566	0.747	0.151	0.801	0.386	0.264
Intra-group comparison^c^	*T*	-1.525	0.969	-0.31	0.55								
	*P*	0.14	0.342	0.759	0.588								
DBP^a^	Before treatment	70.6±10.4	76.1±8.3	72.2±11.5	72.4±9.4	1.316	0.274	0.055	0.333	0.521	0.333	0.196	0.743
	After treatment	74.6±11.5	72.8±10.9	73.3±9.7	72.8±8.8	0.176	0.912	0.53	0.682	0.53	0.827	1	0.827
Intra-group comparison^b^	*T*	-1.413	1.197	-0.369	-0.166								
	*P*	0.171	0.243	0.715	0.87								
BMI^a^	Before treatment	22.94±2.37	23.67±1.98	23.51±2.12	23.78±2.25	0.735	0.534	0.237	0.354	0.177	0.796	0.867	0.67
	After treatment	22.94±2.87	22.86±2.41	23.03±2.88	23.44±1.72	0.26	0.854	0.902	0.906	0.491	0.81	0.417	0.568
Intra-group comparison^c^	*T*	-0.012	1.201	0.627	0.648								
	*P*	0.991	0.241	0.537	0.523								
Respiratory rate^a^	Before treatment	16.2±2.3	15.8±2.5	16.3±2.6	16.2±2.0	0.292	0.831	0.903	0.502	0.903	0.428	1	0.428
	After treatment	16.3±2.1	16.1±2.3	16.0±2.0	15.9±2.6	0.17	0.917	0.586	0.856	0.545	0.716	0.952	0.672
Intra-group comparison^c^	*T*	-0.569	1.995	-1.844	1.565								
	*P*	0.574	0.058	0.078	0.131								
Waist-hip rate^b^	Before treatment	0.88 (0.79, 0.90)	0.86 (0.76, 0.90)	0.80 (0.74, 0.89)	0.86 (0.77, 0.91)	4.119	0.249						
	After treatment	0.87 (0.79, 0.90)	0.86 (0.80, 0.89)	0.81 (0.75, 0.89)	0.87 (0.72, 0.90)	1.066	0.785						
Intra-group comparison^d^	*Z*	-1.674	-0.537	-1.024	-1.183								
	*P*	0.094	0.591	0.306	0.237								
Body temperature^b^	Before treatment	36.70 (36.35, 36.90)	36.60 (36.30, 36.80)	36.60 (36.30, 36.85)	36.60 (36.35, 36.80)	0..586	0.9						
	After treatment	36.30 (36.20. 36.70)	36.40 (36.20, 36.50)	36.40 (36.20, 36.85)	36.60 (36.30, 36.90)	4.957	0.175						
Intra-group comparison^d^	*Z*	-1.985	-2.185	-1.037	-0.384								
	*P*	0.047	0.029	0.300	0.701								

^a^ Data are expressed as the mean ± SD, and were analyzed by one-way analysis of variance followed by least significant difference method. ^b^ Data are expressed as median (interquartile range), and were analyzed by the Kruskal-Wallis rank sum test. The intra-group comparison was performed by paired *t*-test ^c^ or Friedman test ^d^. BG: Blank group; CMG: Chinese medicines group; HCMG: hydrogen-rich water and Chinese medicine group; HG: hydrogen-rich water group. BMI: body mass index; DBP: diastolic blood pressure; HR: heart rate; SBP: systolic blood pressure.

### Effective rate of kidney stone under hydrogen-rich water combined with traditional Chinese medicine treatment

Statistical analysis revealed that the total effective rates of kidney stone treatment in blank group, Chinese medicine group, HRW + Chinese medicine group and HRW group were 28%, 76%, 80% and 32%, respectively. The chi-square test showed that the differences among the four groups were statistically significant (*P* < 0.001). As shown in **Table 3**, the effective rates of Chinese medicine group and HRW + Chinese medicine group were higher than those of blank group and HRW group. Further multiple comparisons showed that the difference was statistically significant (*P* < 0.05). However, the differences between effective rates of Chinese medicine group and HRW + Chinese medicine group, and between blank group and HRW group, were not statistically significant (*P* ≥ 0.05).

**Table 3:** Effective rate of kidney stone of patients in the four groups

	BG	CMG	HCMG	HG	*χ* ^2^	*P*
Recovery	5(20%)	8(32%)	11(44%)	5(20%)	23.35	< 0.001
Effective	2(8%)	11(44%)	9(36%)	3(12%)		
Ineffective	18(72%)	6(24%)	5(20%)	17(68%)		

Data are expressed as the number (percentage), and were analyzed by the chi-square test followed by Bonferroni method. BG: Blank group; HG: hydrogen-rich water group; HCMG: hydrogen-rich water and Chinese medicine group; CMG: Chinese medicines group.

### Urine metabolism under hydrogen-rich water combined with traditional Chinese medicine treatment

As shown in **Table 4** and **Additional Table 2**, the urine test results, including urine metabolic indicators (urine calcium/creatinine, oxalate/creatinine, phosphate/creatinine, uric acid, citrate/creatinine, magnesium/creatinine and PH) and renal injury markers (urine NAG and β2-MG) before treatment showed no difference among the four groups. Compared to before treatment, there was no difference in the urinary calcium/creatinine, oxalate/creatinine, PH and β2-MG after treatment, and there was also no difference among the four groups after treatment. Urine phosphate/creatinine and urine acid had no difference among the four groups after treatment, and compared with pre-treatment, the reduction after treatment was statistically significant only in the HRW + Chinese medicine group (*P* = 0.028). Urinary citrate/creatinine increased in HRW + Chinese medicine group after treatment, and it was significantly different from that in blank group (*P* = 0.019) and before treatment (*P* = 0.004). Urinary magnesium was significantly increased in Chinese medicine group (*P* < 0.001) and HRW + Chinese medicine group (*P* < 0.001) after treatment, and were also increased compared from that in blank group, HRW group. Urine NAG was significantly decreased in Chinese medicine group (*P* < 0.001) and HRW + Chinese medicine group (*P* < 0.001) after treatment, and there were statistically significant differences in Chinese medicine group vs. blank group (*P* = 0.031), HRW + Chinese medicine group vs. blank group (*P* = 0.004), and HRW + Chinese medicine group *vs*. HRW group (*P* = 0.046) after treatment.

**Table 4:** Urine metabolic index of patients in the four groups

Urine metabolic index		BG (*n* = 25)	CMG (*n* = 25)	HCMG (*n* = 25)	HG (*n* = 25)	Intergroup comparison	
						*F*/*H*	*P*
Urine calcium/creatinine^a^ (mmol/mmol)	Before treatment	0.28±0.15	0.24±0.16	0.3±0.19	0.25±0.16	0.614	0.608
	After treatment	0.27±0.19	0.27±0.16	0.26±0.14	0.25±0.18	0.077	0.972
Intra-group comparison^c^	*t*	0.22	–0.678	0.78	–0.054		
	*P*	0.828	0.504	0.443	0.957		
Urine phosphate/creatinine^a^ (mmol/mmol)	Before treatment	1.41±0.61	1.28±0.65	1.56±0.66	1.29±0.57	1.114	0.347
	After treatment	1.48±0.51	1.30±0.60	1.41±0.51	1.28±0.57	0.709	0.549
Intra-group comparison^c^	*t*	–0.881	–0.38	2.342	0.308		
	*P*	0.387	0.708	0.028*	0.761		
Urine acid^a^ (mM)	Before treatment	2.26±1.22	2.02±1.32	2.01±1.01	2.24±1.54	0.287	0.835
	After treatment	2.13±1.13	2.11±1.19	1.60±0.95	1.54±1.16	1.293	0.281
Intra-group comparison^c^	*t*	1.138	–1.125	5.424	1.409		
	*P*	0.266	0.272	< 0.001**	0.172		
Urine citrate/creatinine^a^ (mmol/mmol)	Before treatment	0.17±0.10	0.21±0.13	0.21±0.15	0.18±0.12	0.432	0.731
	After treatment	0.18±0.12	0.21±0.14	0.26±0.12	0.2±0.12	2.032	0.115
Intra-group comparison^c^	*t*	–0.279	–0.428	–3.195	–0.991		
	*P*	0.783	0.627	0.004**	0.332		
Urine magnesium/creatinine^a^ (mmol/mmol)	Before treatment	0.29±0.15	0.23±0.12	0.22±0.13	0.25±0.14	1.224	0.305
	After treatment	0.28±0.13	0.38±0.21	0.41±0.22	0.28±0.13	3.793	0.013*
Intra-group comparison^c^	*t*	0.433	–4.11	–6.944	–1.737		
	*P*	0.669	< 0.001**	< 0.001**	0.095		
Urine NAG^a^ (IU/L)	Before treatment	9.46±3.57	9.17±3.54	10.29±3.37	9.41±3.48	0.491	0.689
	After treatment	10.02±3.94	7.62±4.12	6.82±3.96	9.04±3.49	3.394	0.021*
Intra-group comparison^c^	*t*	–1.479	4.267	4.861	0.948		
	*P*	0.152	< 0.001**	< 0.001**	0.353		
Urine β2-MG^a^ (mg/L)	Before treatment	0.20±0.06	0.21±0.05	0.20±0.07	0.21±0.06	0.171	0.916
	After treatment	0.20±0.06	0.20±0.06	0.19±0.05	0.19±0.05	0.641	0.591
Intra-group comparison^c^	*t*	–0.252	0.866	0.958	1.913		
	*P*	0.803	0.395	0.348	0.068		
Urine PH^b^	Before treatment	6.50 (5.50, 7.00)	6.00 (5.50, 6.50)	6.50 (5.75, 7.00)	6.00 (5.50, 6.50)	2.637	0.451
	After treatment	6.00 (5.50, 7.00)	6.00 (5.50, 7.00)	6.40 (6.00, 7.20)	6.00 (6.00, 6.75)	2.787	0.426
Intra-group comparison^d^	*Z*	–0.51	–0.041	–0.537	–0.761		
	*P*	0.67	0.819	1	0.593		
Urine oxalate/creatinine^b^ (mmol/mmol)	Before treatment	0.03 (0.01, 0.05)	0.03 (0.01, 0.05)	0.03 (0.01, 0.07)	0.03 (0.02, 0.07)	1.314	0.726
	After treatment	0.04 (0.02, 0.08)	0.03 (0.01, 0.07)	0.01 (0.01, 0.06)	0.03(0.01, 0.06)	4.105	0.25
Intra-group comparison^d^	*Z*	–1.674	–0.537	–1.024	–1.183		
	*P*	0.094	0.591	0.306	0.237		

^a^ Data are expressed as the mean ± SD, and were analyzed by one-way analysis of variance. ^b^ Data are expressed as median (interquartile range), and were analyzed by the Kruskal-Wallis rank sum test. The intra-group comparison was performed by paired *t*-test ^c^ or Friedman test ^d^. *P < 0.05, **P < 0.01. BG: Blank group; CMG: Chinese medicines group; HCMG: hydrogen-rich water and Chinese medicine group; HG: hydrogen-rich water group. NAG: N-Acetyl-β-D-glucosaminidase; PH: hydrogen ion concentration; β2-MG: β2-microglobulin.

**Additional Table 2:** Multiple comparisons of urine metabolic index of patients in the four groups

Urine metabolic index		BG *vs.* CMG	BG *vs.* HCMG	BG *vs.* HG	CMG *vs.* HCMG	CMG *vs.* HG	HCMG *vs.* HG
Urine calcium/creatinine (mmol/mmol)	Before treatment	0.38	0.77	0.5	0.25	0.85	0.33
	After treatment	0.97	0.88	0.7	0.85	0.67	0.81
Urine phosphate/creatinine (mmol/mmol)	Before treatment	0.479	0.383	0.496	0.116	0.978	0.122
	After treatment	0.27	0.648	0.2	0.516	0.857	0.407
Urine acid (mM)	Before treatment	0.503	0.484	0.947	0.976	0.546	0.947
	After treatment	0.938	0.09	0.773	0.106	0.833	0.158
Urine citrate/creatinine (mmol/mmol)	Before treatment	0.355	0.344	0.729	0.928	0.562	0.547
	After treatment	0.342	0.019^*^	0.531	0.157	0.745	0.083
Urine magnesium/creatinine (mmol/mmol)	Before treatment	0.162	0.071	0.332	0.679	0.664	0.398
	After treatment	0.04^*^	0.009^**^	0.962	0.56	0.045*	0.01*
Urine NAG (IU/L)	Before treatment	0.765	0.404	0.958	0.258	0.805	0.375
	After treatment	0.031^*^	0.004^**^	0.373	0.466	0.2	0.046^*^
Urine β2-MG (mg/L)	Before treatment	0.593	0.981	0.659	0.577	0.926	0.642
	After treatment	1	0.285	0.392	0.285	0.392	0.83

The least significant difference method is used for multiple comparisons. *P < 0.05, **P < 0.01. BG: Blank group; CMG: Chinese medicines group; HCMG: hydrogen-rich water and Chinese medicine group; HG: hydrogen-rich water group. NAG: N-Acetyl-β-D-glucosaminidase; β2-MG: β2-microglobulin.

### Renal function status under hydrogen-rich water combined with traditional Chinese medicine treatment

As shown in **Table 5** and **Additional Table 3**, there were no differences in serum creatinine before and after treatment among the patients in the four groups. There was no difference in serum uric acid among the four groups before treatment, but there were statistical differences in blank group & Chinese medicine group and blank group & HRW + Chinese medicine group after treatment. Compared with before treatment, serum uric acid statistically decreased in HRW + Chinese medicine group after treatment.

**Table 5:** Blood test of patients in the four groups

Blood index		BG (*n* = 25)	CMG (*n* = 25)	HCMG (*n* = 25)	HG (*n* = 25)	Intergroup comparison	
						*F*	*P*
SCr^a^ (μM)	Before treatment	69.5±16.3	65.6±11.6	68.4±14.1	69.2±15.4	0.371	0.774
	After treatment	69.3±17.8	65.0±11.8	65.9±16.3	68.4±17.7	0.392	0.759
Intra-group comparison^b^	*t*	0.112	0.572	1.593	0.391		
	*P*	0.912	0.573	0.124	0.699		
UA^a^ (μM)	Before treatment	363.7±107.0	338.1±89.0	339.7±84.1	337.4±103	0.431	0.732
	After treatment	379.2±88.7	329.0±86.3	319.1±81.8	346.7±88.8	2.333	0.079
Intra-group comparison^b^	*t*	–1.11	1.054	2.627	–0.933		
	*P*	0.278	0.303	0.015*	0.36		
IL-6^a^ (pg/mL)	Before treatment	8.7±2.3	9.5±2.5	9.4±2.5	8.9±2.7	0.633	0.596
	After treatment	9.2±2.3	9.3±2.7	8.7±2.2	8.7±2.9	0.358	0.784
Intra-group comparison^b^	*t*	–1.58	0.405	1.904	0.486		
	*P*	0.127	0.689	0.069	0.631		
GSH^a^ (U/L)	Before treatment	57±7.3	56.8±8.1	58.5±6.6	58.1±7.4	0.328	0.805
	After treatment	56.8±5.6	61.7±7.5	64.7±6.1	60.6±1.3	6.494	<0.001**
Intra-group comparison^b^	*t*	0.104	–2.128	–4.312	–1.105		
	*P*	0.918	0.044*	< 0.001**	0.28		
SOD^a^ (U/mL)	Before treatment	245±41	245±46	242±48	250±44	0.131	0.942
	After treatment	231±47	243±53	273±47	241±58	3.046	0.032*
Intra-group comparison^b^	*T*	1.327	0.102	–2.744	0.576		
	*P*	0.197	0.919	0.011*	0.57		

^a^ Data are expressed as the mean ± SD, and were analyzed by one-way analysis of variance. ^b^ The intra-group comparison was performed by paired *t*-test. *P < 0.05, **P < 0.01. BG: Blank group; CMG: Chinese medicines group; HCMG: hydrogen-rich water and Chinese medicine group; HG: hydrogen-rich water group. ALT: alanine aminotransferase; AST: aspartate aminotransferase; GSH; glutathione reductase; IL-6: interleukin-6; PLT: platelet; SCr: serum creatinine; SOD: superoxide dismutase; UA: uric acid.

**Additional Table 3:** Multiple comparisons of blood test of patients in the four groups

Blood index		BG *vs.* CMG	BG *vs.* HCMG	BG *vs.* HG	CMG *vs.* HCMG	CMG *vs.* HG	HCMG *vs.* HG
SCr^a^ (μM)	Before treatment	0.35	0.804	0.948	0.491	0.384	0.855
	After treatment	0.348	0.463	0.842	0.837	0.459	0.593
UA^a^ (μM)	Before treatment	0.355	0.381	0.336	0.961	0.971	0.932
	After treatment	0.043^*^	0.016^*^	0.186	0.685	0.472	0.262
IL-6^a^ (pg/mL)	Before treatment	0.233	0.316	0.735	0.848	0.392	0.506
	After treatment	0.87	0.495	0.556	0.398	0.452	0.926
GSH^a^ (U/L)	Before treatment	0.908	0.473	0.596	0.405	0.519	0.851
	After treatment	0.008^*^	<0.001^**^	0.042^*^	0.104	0.516	0.024^*^
SOD^a^ (U/mL)	Before treatment	0.955	0.79	0.728	0.834	0.686	0.54
	After treatment	0.398	0.005^**^	0.479	0.046^*^	0.891	0.033^*^

The least significant difference method is used for multiple comparisons. *P < 0.05, **P < 0.01. BG: Blank group; CMG: Chinese medicines group; HCMG: hydrogen-rich water and Chinese medicine group; HG: hydrogen-rich water group. GSH; glutathione reductase; IL-6: interleukin-6; SCr: serum creatinine; SOD: superoxide dismutase; UA: uric acid.

### Inflammation and oxidative factor indicators under hydrogen-rich water combined with traditional Chinese medicine treatment

As shown in **Table 5** and **Additional Table 3**, there were no differences in interleukin-6 before and after treatment among the patients in the four groups. GSH had no difference in each group before treatment, but it increased to different degrees after treatment. The increase in Chinese medicine group (*P* = 0.044) and HRW + Chinese medicine group (*P* < 0.001) was statistically significant compared with those before treatment. After treatment, the GSH value of each group was significantly different from that of blank group (*P* < 0.001), and the difference between HRW + Chinese medicine group and HRW group was statistically significant (*P* = 0.024). There was no difference in superoxide dismutase among the groups before treatment, and only the HRW + Chinese medicine group statistically significantly increased after treatment, which was higher than that of pre-treatment (*P* = 0.044) and other groups after treatment (*P* = 0.032).

### Safety under hydrogen-rich water combined with traditional Chinese medicine treatment

There was no difference in safety indicators, including blood routine and liver function, between the groups before and after treatment, and there was no change after treatment (**Additional Table 4**).

**Additional Table 4:** Safety index of patients in the four groups

Safety index		BG (*n* = 25)	CMG (*n* = 25)	HCMG (*n* = 25)	HG (*n* = 25)	Intergroup comparison		*P*					
	
						*F*	*P*	BG *vs.* CMG	BG *vs.* HCMG	BG *vs.* HG	CMG *vs.* HCMG	CMG *vs.* HG	HCMG *vs.* HG
WBC^a^ (×10^9^/L)	Before treatment	6.29±1.29	5.85±1.16	6.23±1.51	6.34±1.39	0.639	0.592	0.523	0.709	0.505	0.313	0.193	0.768
	After treatment	5.76±1.33	5.59±1.53	5.76±1.65	5.84±1.31	0.127	0.944	0.69	0.987	0.844	0.678	0.551	0.857
Intra-group comparison^b^	*t*	0.849	0.643	1.104	1.655								
	*P*	0.404	0.526	0.28	0.111								
RBC^a^ (×10^12^/L)	Before treatment	4.81±0.51	4.66±0.56	4.59±0.67	4.85±0.65	1.025	0.385	0.367	0.202	0.831	0.706	0.266	0.138
	After treatment	4.93±0.57	4.63±0.56	4.67±0.73	4.84±0.65	1.318	0.273	0.087	0.142	0.604	0.804	0.229	0.339
Intra-group comparison^b^	*t*	-0.89	0.242	-0.372	0.041								
	*P*	0.382	0.811	0.713	0.968								
HGB^a^ (g/L)	Before treatment	142±13	143±18	137±18	144±15	0.946	0.421	0.825	0.258	0.703	0.177	0.873	0.132
	After treatment	142±16	139±12	141±11	142±18	0.193	0.901	0.506	0.814	0.984	0.667	0.518	0.829
Intra-group comparison^b^	*t*	0.115	0.991	-0.9	0.533								
	*P*	0.91	0.332	0.377	0.599								
PLT^a^ (×10^9^/L)	Before treatment	200±45	204±55	199±54	204±52	0.049	0.986	0.827	0.939	0.816	0.768	0.989	0.757
	After treatment	211±39	201±60	220±57	192±37	1.398	0.248	0.486	0.558	0.185	0.201	0.526	0.057
Intra-group comparison^b^	*t*	-0.87	0.16	-1.32	0.969								
	*P*	0.393	0.874	0.199	0.342								
ALT^a^ (U/L)	Before treatment	30.9±11.5	29.1±10.2	30.9±11.8	30.7±11.7	0.147	0.931	0.576	0.998	0.943	0.574	0.625	0.941
	After treatment	26.2±7.9	28.4±10.2	30.1±11.2	30.0±12.9	0.718	0.544	0.461	0.206	0.211	0.595	0.606	0.987
Intra-group comparison^b^	*t*	1.684	0.213	0.251	0.175								
	*P*	0.105	0.833	0.804	0.863								
AST^a^ (U/L)	Before treatment	23.1±10.9	24.2±6.6	21.7±7.7	25.0±7.6	0.726	0.539	0.639	0.562	0.423	0.295	0.739	0.169
	After treatment	25.3±9.4	24.9±7.8	23.4±7.9	22.8±7.0	0.558	0.644	0.849	0.406	0.265	0.521	0.355	0.776
Intra-group comparison^b^	*t*	-0.657	-0.335	-0.808	1.049								
	*P*	0.517	0.74	0.427	0.305								

^a^ Data are expressed as the mean ± SD, and were analyzed by one-way analysis of variance followed by least significant difference method. ^b^ The intra-group comparison was performed by paired *t*-test. BG: Blank group; CMG: Chinese medicines group; HCMG: hydrogen-rich water and Chinese medicine group; HG: hydrogen-rich water group. ALT: alanine aminotransferase; AST: aspartate aminotransferase; HGB: hemoglobin; PLT: platelet; RBC: red blood cell; WBC: white blood cell.

### Traditional Chinese medicinal syndrome score under hydrogen-rich water combined with traditional Chinese medicine treatment

First, the Mauchly’s sphericity test showed that the data met the sphericity assumption (*W* = 0.974, *P* = 0.783). Descriptive statistics indicated that, the mean values of the four groups at each time point showed an upward trend (**Table 6**). The main effect of time was significant (*F* = 41.525, *P* < 0.001), indicating that the measured value changed significantly with time. The interaction effect between group and time was significant (*F* = 7.307, *P* < 0.001), indicating that there were differences in the trend of change over time among the four groups. The pairwise comparison of the main effect of time with Bonferroni correction found that there was no statistical difference in traditional Chinese medicinal syndrome score among the four groups at week 0, and the difference in traditional Chinese medicinal syndrome score between the groups started to be statistically different from week 4. In conclusion, the three groups of different interventions except blank group can reduce the traditional Chinese medicinal syndrome score, and the effect continues to enhance over time. This reduction mode has no differences between blank group & HRW group and Chinese medicine group & HRW + Chinese medicine group.

**Table 6:** TCM syndrome score of patients in the four groups

Time point	BG	CMG	HCMG	HG	*F*	*P*
0 wk	24.56±2.92	24.32±3.69	24.8±2.66	24.8±2.2	0.15	0.926
4 wk	23.92±2.8	21.84±2.94	22.28±2.49	24.48±2.97	5.11	0.003
8 wk	24.2±2.99	21.2±2.47	19.92±1.44	22.48±2.82	13.37	< 0.001
12 wk	22.36±3.89	18.44±3.34	18.28±2.05	22.04±3.17	12.16	< 0.001

*F*	2.472	15.919	22.89	5.809		
*P*	0.67	< 0.001	< 0.001	0.001		

Data are expressed as the mean ± SD, and were analyzed by repeated measures analysis of variance. *F*_time_ = 41.525, *P*_time_ < 0.001; *F*_group_ = 19.742, *P*_group_ < 0.001; *F*_group*time_ = 7.307, *P*_group*time_ < 0.001. For *P*_group*time_ < 0.001, simple effect analysis and pairwise repeated comparison were performed. BG: Blank group; CMG: Chinese medicines group; HCMG: hydrogen-rich water and Chinese medicine group; HG: hydrogen-rich water group.

## Discussion

In this study, we evaluated the effectiveness of YSSLF combined with HRW therapy, improving abnormal urinary metabolism in patients with kidney stones. Our findings indicated that the YSSLF treatment exhibited improvements in total effective rate in kidney stone treatment and related traditional Chinese medicinal syndrome score, it can partially alleviate the oxidative stress, uric acid metabolism and urinary magnesium levels, and improve the function of renal tubules of patients with kidney stones. The HRW therapy showed only efficacy in improving GSH, but the combination of YSSLF and HRW has shown superior efficacy in improving oxidative stress and related metabolic factors of blood uric acid and urine stones, as well as in renal tubular function. The novelty and significance of our study lie in its exploration of the therapeutic benefits of traditional Chinese medicine combined with HRW beyond its established discharging stone effects. Our research has introduced a completely new integrated treatment approach combining traditional Chinese medicine and H_2_ for kidney stones.

An epidemiological study has shown that patients with metabolic syndrome are at risk of nephrolithiasis.^17^ Metabolic syndrome^18^ and metabolic derangement^19^ both play a significant role in stone formation. The correlation between metabolic changes and patients with kidney stones has become a research hotspot in recent years. Metabolic abnormalities were found in 91% of young and adult patients with renal lithiasis.^20^ Low urine volume, high urinary excretion of calcium, phosphates, oxalates, uric acid and cysteine are considered as promotors.^21^ Citrate, magnesium and other organic substances (nephrocalcin, urinary prothrombin fragment-1, and osteopontin) are known to inhibit stone formation.^22^ Patients with kidney stones are also more likely to have urinary metabolic abnormalities compared to healthy individuals,^23^ while patients with recurrent stones tend to have more significant metabolic abnormalities than those with a single stone episode.^24^ According to the European Association of Urology Guidelines, metabolic stone evaluation is highly recommended to prevent stone recurrences.^5^

Natural active ingredients in traditional Chinese medicine exhibit characteristics of being multi-component, multi-target, and multi-pathway properties. Interactions among these components can lead to therapeutic efficacy. In the preliminary research, we found that both traditional Chinese medicinal compound formula^25^ and single herb^26^ have curative effects in kidney stones. In this study, we observed that the YSSLF treatment can improve urine magnesium/creatinine, while YSSLF combined with HRW treatment can simultaneously improve urine citrate/creatinine, reduce urine phosphate/creatinine and urine acid. Magnesium decreases both calcium oxalate and calcium phosphate aggregation in a concentration-dependent manner.^27^ Magnesium also works as a chelator of oxalate in the bowel, thus reducing its intestinal absorption.^28^ Citrate has a high affinity for calcium, inhibiting crystallization of calcium crystals and it also prevents the aggregation of already formed calcium oxalate crystals, thus preventing the formation of bigger concretions and stones.^28^ This indicates that YSSLF may play a role in the treatment of kidney stones by improving the protective factors in urine, and the combination of YSSLF and HRW will strengthen the improvement of protective components in urine, reduce the risk factors of urinary stones, improve the treatment effect of stones, and may even reduce the recurrence of stones.

Oxalate in urine could cause oxidative damage to renal epithelial cells and increase intracellular ROS levels.^29^ Oxidative stress damage to renal epithelial cells is the main pathological factor of calcium oxalate calculi formation.^30^ Renal exposure to oxalates could cause lipid peroxidation and produce ROS, followed by renal cell injury and inflammation. The resultant loss of membrane integrity promotes fibrosis and collagen formation, facilitates calcium oxalate adhesion, retention and subsequent stone formation.^31^ The development of medicine that could alleviate oxidative damage has become the key to the prevention and treatment of urolithiasis.^30^ The anti-inflammatory and antioxidant effects of H_2_ have been confirmed.^32^ Both H_2_ inhalation^33^ and HRW^34^ may alleviate oxalate-induced kidney injury with their anti-oxidative, anti-inflammatory and anti-fibrotic effects. In calcium oxalate-induced renal injury, urine metabolites showed significant restoration to normal levels after H_2_ treatment.^33^ Traditional medicinal plants also have antioxidant and anti-inflammatory effects.^10^

Urinary NAG exists in the lysosomes of proximal tubule epithelial cells and increased NAG excretion in urine suggested proximal renal tubular dysfunction. A previous study has shown that, there was a significant association between bilateral renal stones and kidney tubular injury markers, like urinary NAG/creatinine.^35^ A case-control study of children showed that NAG/creatinine ratios in urolithiasis and nephrocalcinosis patients were significantly higher than those in healthy controls.^36^ In this study, the simultaneous use of YSSLF and HRW showed better antioxidant effects and less renal tubular damage, suggesting that the combined therapy may have a better therapeutic effect on stone related renal tubular injury, stone formation and recurrence, and it should be clarified in further experiments.

This study has several limitations. Firstly, due to the traditional Chinese medicinal drug formulation and HRW preparation, the subject did not undergo blinding in this study, which may affect the results. Future studies should consider improving the delivery method of H_2_ or employing independent observers to minimize such biases. Secondly, the single-center design of our study may limit the generalizability of our findings. To increase the external validity and broader clinical applicability of our results, we recommend that future studies adopt a multicenter approach.

In summary, although in combination with HRW and YSSLF cannot improve the therapeutic effect of kidney stones, the addition of HRW can improve urinary metabolism and oxidative stress status in the treatment of kidney stones with YSSLF, thereby enhancing its role in kidney protection and stone recurrence.

## Additional files:

***Additional file 1:*** *CONSORT 2025 checklist.*

***Additional Table 1:*** *General characteristics in the four groups.*

***Additional Table 2:*** *Multiple comparisons of urine metabolic index of patients in the four groups.*

***Additional Table 3:*** *Multiple comparisons of blood test of patients in the four groups.*

***Additional Table 4:*** *Safety index of patients in the four groups.*

## Data Availability

*All data relevant to the study are included in the article or uploaded as additional files.*
